# A Natural Experiment Created by Pandemic Restriction: Comparing In-Person, Hybrid, & Teletherapy Formats

**DOI:** 10.1093/geroni/igab046.2949

**Published:** 2021-12-17

**Authors:** Tracy Wharton, Daniel Paulson

**Affiliations:** 1 University of Central Florida, Winter Springs, Florida, United States; 2 University of Central Florida, Orlando, Florida, United States

## Abstract

The FL-REACH intervention for families in early stage post-dementia diagnosis was originally adapted from the REACH II program for use in an outpatient clinic. Pandemic restrictions forced an adaptation to a teletherapy format. The timing of changes allowed comparison of caregivers who participated in clinic (n=10), switched modalities mid-treatment (n=7), and participated as teletherapy (n=14). Groups were similar in age range, gender, and relationship, with both spouses and adult children participating. Participants in the fully online group were more likely than others to have high school or trade school education than to have graduated from college. All participants in the in-person and hybrid groups had incomes over $40,000/year, while 36% of the online sample had less household income, were more likely to be Hispanic-identifying (43% versus 6%), and had higher burden scores (M=41.43 versus M=32.56 in person, M=29.86 hybrid) and lower preparedness scores (M=19.86 versus M=22.90 in person, M=28.14 hybrid) at baseline (p<.05). The intervention proceeded with the same intervention dosage (8 hours total), and outcomes were essentially comparable, with all groups showing statistically significant improvement on measures of preparedness, burden, and risk. While in-person intervention helped strengthen relationships with the medical team, inclusion of family via telehealth provided opportunity for a more culturally responsive and inclusive engagement, although there remain questions regarding reasons for differences at baseline. Identification of differences in key outcomes for direct comparisons between in-person, hybrid, and teletherapy interventions are limited in the evidence base, making this a unique study at an important moment in time.

